# Efficacy and tolerability of oral gastrodin for medication overuse headache (EASTERN): Study protocol for a multicenter randomized double-blind placebo-controlled trial

**DOI:** 10.3389/fneur.2022.1095298

**Published:** 2023-02-22

**Authors:** Fanyi Kong, Dawn C. Buse, Jia Geng, Jingjing Xu, Hanxiang Liu, Shu Ma

**Affiliations:** ^1^Department of Neurology, The Affiliated Hospital of Yunnan University, Kunming, Yunnan, China; ^2^Department of Neurology, Albert Einstein College of Medicine, Bronx, NY, United States; ^3^Department of Neurology, Affiliated Hospital and Clinical Medical College of Chengdu University, Chengdu, Sichuan, China; ^4^Department of Neurology, Xiangya Changde Hospital, Changde, Hunan, China; ^5^Department of Neurology, Puer People's Hospital, Puer, Yunnan, China; ^6^Department of Neurology, 920th Hospital of Logistics Support Force, People's Liberation Army, Kunming, Yunnan, China

**Keywords:** medication overuse headache, gastrodin, randomized controlled trial, detoxification, prophylaxis

## Abstract

**Background:**

Prophylactic medication in clinical detoxification programs for the treatment of medication overuse headache is still debated. Gastrodin, a main bioactive constituent of *Rhizoma Gastrodiae*, has been applied clinically to treat primary headache for more than 30 years in China due to its potential analgesic and anti-migraine mechanisms. However, clinical evidence supporting its routing use in MOH is insufficient. The present study aims to investigate the efficacy and tolerability of oral gastrodin in medication overuse headache.

**Methods:**

A multicenter, randomized, double-blind, parallel, placebo-controlled trial will be performed. A target sample size of 186 patients who fulfill the International Classification of Headache Disorders 3rd version (ICHD-3) criteria for MOH will be recruited and screened during a baseline screening period of 28 days before being randomly assigned to either the gastrodin or placebo group at a ratio of 1:1. Enrolled patients will be assessed for each 4 weeks during the 12-weeks double-blind phase and followed up at week 24. The primary endpoint is mean change in monthly headache day frequency. Secondary endpoints will be the proportion of remitted MOH, change in headache pain intensity, headache impact test (HIT-6) score, 50% responder rate, treatment failure, monthly acute medication intake days, and Short Form 36-Item Health Survey (SF-36) score. Tolerability will be assessed by drop-out rates though safety monitoring during treatment.

**Discussion:**

The findings of the present study may help to provide new evidence on gastrodin as a prophylaxis treatment with both efficacy and high tolerability for the treatment of MOH.

**Clinical trail registration:**

Chinese Clinical Trail Registry (ChiCTR2200063719), Protocol Version 1.1, May, 09, 2022.

## Introduction

### Background and rationale

Medication overuse headache generally refers to monthly headache days of ≥15 per month resulting from acute “medication overuse” (MO) defined as the frequent consumption of acute pain medications above a certain threshold (e.g., 10 or 15 days per month depending on the medication type) for ≥3 months. Diagnostic criteria for MOH were introduced in the International Classification of Headache Disorders, 2nd edition (ICHD-2) (1) without a the requirement of the diagnosis of chronic migraine (CM; see Appendix for details on MOH and CM). In the third edition beta version (ICHD-3β), updated in 2013, concurrent CM and MOH are diagnosed if both criteria are met (2). These criteria remain the same in the ICHD-3 criteria.

The prevalence of MOH is still uncertain (3) and varies from 2% in a recent population-based study (4) to 60% in the Global Burden of Disease Study (5). Whether MOH is a cause or consequence of frequent acute medication intake has been debated for years (6, 7). Nonetheless, MO and MOH management are important in clinical care. MO is associated with an increased risk of chronic headache (8) and chronic migraine (9, 10).

Prognosis of MOH due to different type of chronic headache is probably variable. Although there is evidence that chronic tension-type headache (CTTH) and CM may have partly distinct pathophysiological mechanisms, clinical experience suggests that these two clinical entities may be at opposite poles of a single disease spectrum (11, 12). Of note, CM with MO represents a more severe subgroup of patients and can be difficult to treat (13). In a preplanned subgroup analysis that evaluated efficacy and safety of erenumab in patients with CM and MO, the medication overuse subgroup had a higher proportion of failing to respond to preventive treatment (14).

Effective education for people with MOH plays a major role in effective withdrawal therapy. Successful educational interventions rely largely on a productive doctor-patient alliance (15) and patients' motivation for adherence. In addition, it is often recommended to add a preventive therapy or optimize existing preventive regimens to reduce the number of monthly headache days, improve sense of self efficacy and as a result reduce both the physical and psychological need for acute medication for migraine. Preventive treatments may be pharmacologic, non-pharmacologic, or both.

Moreover, the addition of behavioral therapies to medication withdrawal has been shown to significantly improve outcomes and the maintenance of benefits when measured at 3 years post treatment) (16, 17). Results from a previous systematic review (18) and a newly published randomized clinical trial (19) support a program of prophylaxis treatment together with abrupt withdrawal of acute analgesic intake for treating MOH. Evidence from randomized controlled trials confirms complete withdrawal of the offending acute medication(s) is an effective treatment for MOH (20–22), but withdrawal symptoms may lead to treatment failure or rebound reactions. In the past two decades, it has been suggested that prednisone may be effective in reducing withdrawal symptoms (23, 24). However, results from a well-designed controlled trial confirmed no therapeutic effect of prednisolone on MOH (25). Therefore, an acceptable level of evidence in the recommending use of any of prophylactic medications or treatments other than behavioral therapies is still undetermined up to now (26). Hence the utility in bridging therapy using a preventive drug during the withdrawn phase needs to be determined. Topiramate is the first choice for people with MOH despite frequent adverse events (27). Data from our ongoing network meta-analysis (PROSPERO, CRD 42021193370) indicate that despite lower safety and greater tolerability issues, topiramate has large beneficial effects on reducing headache frequency and monthly acute medication intake frequency. Onabotulinumtoxin A and the humanized monoclonal calcitonin gene-related peptide (CGRP) antagonists eptinezumab and fremanezumab, are also helpful for reversion to no medication overuse; however, until CGRP antagonists are readily available around the world there are challenges with cost-effectiveness and accessibility.

*Rhizoma Gastrodiae*, a kind of traditional Chinese herb medicine, was firstly recorded for treating primary headache in a famous compendium of Chinese medicines called *Sheng Nong's Herbal Classic* in 25 AD to 220 AD. Gastrodin (4-[β-D-glucopyranosyloxy] benzyl alcohol) is the main bioactive constituent of *Rhizoma Gastrodiae* and has been applied clinically as an analgesic to treat migraine and other types of headache for more than 30 years in China (28). Several potential anti-migraine mechanisms of gastrodin have been discovered including inducing a dose-dependent reduction of CGRP- mRNA expression similar to flunarizine (29), preventing the release of CGRP from pre-synaptic central projections, inhibiting the post-synaptic effects of the ERK1/2 downstream signaling pathway to inhibit the firing of second-order trigeminal nociceptive neurons (30), altering acid-evoked membrane excitability of rat dorsal root ganglion neurons and decreasing the amplitude of the depolarization (31). Gastrodin has determined to be safe for use in toxicity studies in animals and occasionally reported adverse drug reactions (ADR) or events (ADE) in clinical practice (32). Recommended dosage of oral gastrodin for treating episodic migraine is 300 mg/day. Although there is no evidence that gastrodin is superior to other analgesics in treating primary headache, low quality evidence obtained from the tremendous amount of case series and observational studies indicates that gastrodin is beneficial for reducing headache frequency and headache intensity with increased dosage. This may largely be explained by the phenomenon that gastrodin is not brain penetrant with a cerebrospinal fluid /plasma ratio of only 5.5% (33). So theoretically, increasing the dosage of gastrodin maybe an ideal option for better efficacy. Previously, the safety of intravenous 40 mg/kg (up to 2,400 mg/day for a 60 kg human) gastrodin had been determined in a clinical trial (34). For efficacy and safety concern, the dosage of oral gastrodin in the present trial will be increased to 600 mg/day.

### Objectives

We will test the hypothesis that gastrodin may serve as a candidate therapy drug during the detoxification phase of MOH with advantages of improving chances of treatment adherence and success. The present study aims to determine the efficacy and tolerability of oral gastrodin as a prophylaxis treatment in MOH.

### Trial design

**Figure 1:**
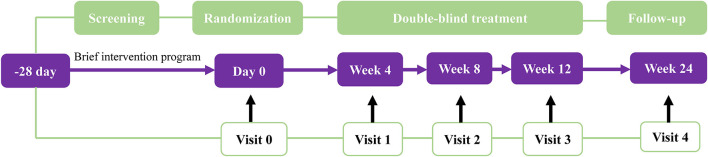
Schematic diagram of study design.

This is an investigator-initiated, multicenter, 1:1 ratio randomized, double-blind, parallel-group, placebo-controlled trial which consisted of a 28-day screening period, and a 12-week intervention period, with a final evaluation at week 24. A schematic diagram of study design is provided in Figure 1. The protocol of the present study has been registered in the Chinese Clinical Trial Registry (ChiCTR2200063719).

## Methods: Participants, interventions, and outcomes

### Study setting

Eligible patients will be screened at neurological outpatient clinic from three study sites at different provinces in China: Affiliated hospital of Yunnan University, Kunming, Yunnan; Xiangya Changde Hospital, Changde, Hunan; and Affiliated hospital of Chengdu University, Chengdu, Sichuan; and Puer People's Hospital, Puer, Yunnan. These sites are all tertiary hospitals which may provide extensive healthcare service for persons with headaches.

### Eligibility criteria

#### Inclusion criteria

Eligible patients are men or women aged 18–70 years with a pre-existing headache disorder onset at or before age 50, fulfilling the ICHD-3 diagnostic criteria for MOH (coded as 8.2) (35) with ≥15 headache days per month and with regular overuse of acute pain medications ≥ 10 or 15 days per month depending on the medication type for ≥ 3 months.

#### Exclusion criteria

Patients who previously received onabotulinum toxin A, nerve block anesthesia or transcranial magnetic stimulation during the 4 months before screening, who are unable to provide headache diary during the screening period, who have history of stroke, epilepsy or receiving antiepileptic drugs, and females who are breast feeding, pregnant or planning a pregnancy during the study will be excluded.

### Interventions

All patients will receive full individualized brief intervention (BI) education according to Kristoffersen et al.'s strategy (36) before randomization.

In the gastrodin group, patients will receive gastrodin 600 mg/day (200 mg tid) from baseline to the end of week 12. In the placebo group, matching placebo made of starch will also be prescribed three times a day. If a patient experience unacceptable adverse events as recorded in the instructions for gastrodin such as dry mouth, dizziness and upper abdominal discomfort, the target dose will be tapered down to 300 mg/day.

During the 24-week study period, their usual acute medication intake will be restricted to ≤ 2 days per week. Participants will be asked to return the experimental drug (or placebo) packaging at week 4, and week 8 in the clinic in order to get drugs for the next 4 weeks.

### Outcome measures

As there is a lack of guidelines for reporting results from clinical trials on MOH, we will adopt Hagen et al.'s suggestion on endpoints for MOH studies in follow-up including headache days/month, acute medication use days/month, headache intensity, cured MOH, and responder rate (37).

Primary efficacy endpoint is reduction in the mean number of monthly headache days from baseline at 24-week follow-up.

Secondary efficacy endpoints include:

a. Headache intensity at 12 and 24-week follow-up. The headache intensity at each headache onset will be measured on a 4-point scale (0 = pain, 1 = mild pain, 2 = moderate pain, 3 = severe pain), and be calculated as the average of headache intensity at each headache onset for 1 month;
b. Proportion of cured MOH, defined as a patient does not fulfill the diagnostic criteria for MOH in ICHD-3 at 12 and 24-week follow-up;
c. Mean change from baseline in total HIT-6 score and the proportion of patients with a ≥ 5-point reduction in total HIT-6 score (38) at 12 and 24-week follow-up. For total HIT-6 scores, a between-treatment group minimally important difference (MID) has been established as ≥2.3 (39). Additionally, a clinically meaningful change for an individual patient has been defined as a ≥ 5-point decrease in total HIT-6 score;
d. 50% responder rate, defined as 50% reduction of from baseline in number of moderate or severe headache days at 12- and 24-week follow-up;
e. Treatment failure, defined as use of acute medication intake >2 days per week during 12-week treatment;
f. Reduction in the mean number of monthly medication intake days for treatment failures at 12- and 24-week follow-up;
g. Quality of life (QoL), as measure by the Short Form 36-Item Health Survey (SF-36) (40) at 12- and 24-week follow-up.

### Safety measures

All subjective adverse events (41) will be collected from the time of allocation through the last visit (week 24). The investigators are responsible for assessment of the severity and causal relationship of all AEs to study intervention. Documentation on start date and duration of AE, treatment measures taken for the AE will be recorded.

Tolerability will be assessed by proportion of drop-outs due to AEs throughout double-blind period, with lower dropout rates indicate higher tolerability.

### Participant timeline

Eligible participants will be enrolled from January, 2023 to December, 2024. During the 28-day screening period, baseline information on demographics, number of monthly headache days, number of monthly acute medication intake days, headache intensity, and sum scores of HIT-6 and SF-36 for the past 4 weeks before randomization will be collected through a patient's headache diary or face to face interview in outpatient clinic. Education on medication overuse using BI strategy (36) will be finished before randomization (Visit 0). Training on the eDiary will be started after a signed consent form obtained from a study participant and prior to the first administration of study intervention. At each visit during the double-blind phase and follow-up (Visit 1–4), outcomes and adverse events will be evaluated correspondingly. Schedule of enrollment, interventions, and assessments following SPIRIT 2013 statement (42) is provided in Table 1.

**Table 1:** SPIRIT flow diagram of enrollment, interventions, and assessments.

**Time point**	**Study period**					
	**Baseline screening**	**Allocation**	**Double-blind treatment period**			**Follow-up**
	**Day-28–0**	**Day 0**	**Week 4 (**±**3 day)**	**Week 8 (**±**3 day)**	**Week 12 (**±**3 day)**	**Week 24 (**±**3 day)**
**Enrollment**						
Eligibility screening	x					
Informed consent	x					
eDiary training	x					
Allocation		x				
BI education	x					
**Interventions**						
Gastrodin			♦———————————————————————–♦			
Placebo			♦———————————————————————–♦			
**Assessments**						
Demographic information	x					
Monthly headache days	x		x	x	x	x
Month medication intake days	x		x	x	x	x
Headache intensity	x		x	x	x	x
Moderate or severe headache days	x		x	x	x	x
Headache Impact Test-6	x		x	x	x	x
Short Form 36-Item Health Survey	x		x	x	x	x
Drop-outs			x	x	x	
Adverse events			x	x	x	

### Sample size

We will estimate each of the above mentioned variables to calculate endpoints for MOH studies according to a published study (22). Hypothesis test will be 2-tailed with 90% power and a significance level of *P* < 0.05. Sample size estimations for the four recommended endpoints are provided in Table 2.

**Table 2:** Sample sizes estimation for each critical endpoint.

**Endpoints**	**Target power**	**Alpha**	**N1**	**N2**	**N**	**Assumed proportion (or mean) for treatment group**	Referred proportion (or mean ±SD) for control group^*^	**Difference between groups**
Reduction in monthly headache days	90%	0.05	77	77	154	46%	22%	24%
Cured medication overuse headache	90%	0.05	44	44	88	90%	62%	28%
Headache intensity	90%	0.05	22	22	44	20.7	38.4 ± 17.4	17.7

* Data referred for sample size estimation are all obtained from a published study (reference 16) which enrolled patients with medication overused headache who were assigned to restricted acute medication intake group (allowing to take up to 2 days a week with analgesics or migraine medication during the study period).

Finally, we assume group sample sizes of 77 in gastrodin group and 77 in the control group will achieve 90.188% power to detect a difference between the group proportions of 24%. The proportion in the gastrodin group is assumed to be 22% under the null hypothesis and 46% under the alternative hypothesis. The proportion in control group is 22% based on previously published literature (22). The test statistic used is the two-sided *Z*-Test with unpooled variance. To allow for 20% dropouts, we will include 186 patients, corresponding to 93 in each group.

## Methods: Assignment of interventions

### Allocation

#### Sequence generation

The external block randomization process will take place at Yunnan University. The project statistician will create a randomized treatment allocation schedule using SPSS by generating 186 numbers. Patients who fulfill criteria for enrollment will be randomly assigned to receive gastrodin or placebo according to a centralized randomization schedule in blocks of 4, stratified by gender and headache frequency at baseline (<14 vs. >14 days per month), to achieve between-group balance in the baseline. Subjects will be assigned to the next available medication number within the block.

#### Allocation concealment mechanism

A designated pharmacist, the only person who has access to the randomization list, will provide the investigators at each center with an opaque vial containing the study medication labeled with the patient's sequential identification number from the randomized allocation schedule. Patients, investigators, the sponsor, and trial staff are unaware of the trial-group assignments.

#### Implementation

An independent research nurse in each center prepares the appropriate treatments. The study investigators enroll participants, administer treatment and assess the safety and outcomes.

### Blinding

The patients, nurses, study investigators and statistician are blinded to the result of the randomization process until data analyses are finished. The blind code could be broken by the principal investigator only for safety concerns. Gastrodin tablets and matching placebo will be provided in identical blister cards to maintain blinding of the study.

## Methods: Data collection, management, and analysis

### Data collection methods

Baseline survey and efficacy assessments will be based on information recorded by participants using an eDiary mobile application. Baseline information includes age, educational level (years), any medical history, family history of headache, body mass index, age of headache onset, pre-exiting headache type, monthly headache days, and month medication intake days.

Following variables will be recorded both at baseline survey and each headache attack after randomization: headache characteristics, headache associated symptoms, the headache pain intensity, pain duration of an attack (hours), and acute medication use (type and dosage).

Monthly headache days, month medication intake days, the headache pain intensity, score of HIT-6 and SF-36, treatment failure, drop-outs and any adverse events will be assessed at 4, 8, 12, and 24-week follow-up.

A summary table of eDiary is shown in Table 3. Training for the eDiary will be provided for qualified participants during before randomization.

**Table 3:** Summary of data collection from eDiary.

**Variables recorded**	**Day-28–0**	**At each headache onset after randomization**			
Age	x				
Body mass index	x				
Education years	x				
Age of first headache onset	x				
Any medical history	x				
Family history of headache	x				
Diagnosis of pre-existing headache	x				
Monthly headache days	x				
Month medication intake days	x				
Headache pain characteristics	x	x	x	x	x
Headache associated symptoms	x	x	x	x	x
Headache intensity	x	x	x	x	x
Duration of headache pain (hours)	x	x	x	x	x
Type of acute pain medication intake	x	x	x	x	x
Dosage of acute pain medication intake	x	x	x	x	x

### Data management

All the data will be downloaded from a cloud server after each visit and recorded in the CRF and then entered into SPSS software (version 20.0) by an independent data administrator. Data on identification information from participants will be de-identified in SPSS database.

### Statistical methods

All analyses on efficacy endpoints will be performed using the intention-to-treat (43) principle which includes all randomized patients who had an evaluable baseline period of eDiary data (week-0), received at least 1 dose of gastrodin or placebo, and had at least one evaluable follow-up appointment. Safety evaluations will be performed consisting of all participants who received at least 1 dose of study intervention.

Continuous variables are presented as mean ± standard deviation (44) conforming to the normal distribution and median (interquartile range; IQR) for the skew distribution data. Student's *t*-test and Mann-Whitney *U*-test will be used to test differences in continuous variables where appropriate. Categorical variables will be expressed by the number and percentage of occurrences. Differences between two groups will be compared using chi-squared tests or Fisher's exact test where appropriate.

Cox proportional hazard regression (backward stepwise method) analysis and the Kaplan-Meier survival curve will be performed when necessary.

To more completely understand the clinical meaningfulness of the new therapy for MOH results from different headache type, we will conduct a subgroup analysis based on prior headache type (CM/CTTH or CM/ other types of headaches) for all the efficacy endpoints.

Participants who do not complete or are lost to follow-up will be coded as treatment failure, missing values will be handled using multiple imputation method under the missing-at-random (MAR) assumption (45).

## Methods: Monitoring

### Data monitoring and harms

An independent data monitoring committee (DMC) will be established to review any spontaneously reported adverse events and identify any safety issues, analyze unintended effects of the study intervention, make recommendations to sponsors, including modification or early termination of the trial when needed. Monitoring on safety trends will be implemented through timely recorded data in eDiary. Other data review on safety issues and pertinent details will be checked by DMC after each visit through source documents. Source documents include a participant's medical records, the eDiary records, and the results of diagnostic tests such as laboratory and neuroimaging tests.

### Auditing

An authorized representative of sponsors will conduct online monitoring to review, audit and download copy of study-related documents on a periodic basis. The representative will meet with the investigator(s) in-site to discuss study-related questions at mutually convenient times when needed.

## Ethics and dissemination

### Research ethics approval

The present study protocol was approved by Ethic Committee of Affiliated Hospital of Yunnan University (No. 2022047).

### Protocol amendments

All modifications to the protocol will be submitted in consultation with Institutional Review Board approval. Summaries of all relevant modifications will be disseminated to each study investigator *via* e-mail in time.

### Consent or assent

Signed consent form including consent provisions for collection and use of participant data and biological specimens will be obtained from participants and authorized surrogates before baseline screening.

### Confidentiality

Data confidentiality will be implemented by identifying ID numbers for all participants, with all data stored and managed by sponsors in a secure. When data sharing is requested, de-identified data files will be transferred on an encrypted mobile storage by sponsors.

### Declaration of interests

Dr. Fanyi Kong was supported by Yunnan Fundamental Research Project (Grant No. 202001AT070127).

### Access to data

All the investigators, including the project statistician will have access to the final trial database after study results are published. Dataset on clinical information is available with the permission of the principal investigators after all the study results are published.

### Ancillary and post-trial care

Compensation to those who suffer harm from trial participation will be covered by commercial insurance provided by KPC Pharmaceuticals, Inc. Free medical services will be provided by Affiliated Hospital of Yunnan University to those who suffer harm from trial participation.

### Dissemination policy

Study findings will be disseminated to healthcare academia through abstracts, posters, or presentations at local, national and international conferences. Full-texts of the present protocol and study results will be published in peer-reviewed journals. Citations of the study findings by professionals are allowed without permission. Participant-level dataset will be provided to professional authors only for conducting individual patient meta-analysis.

## Discussion

Findings from the present study may help to provide an evidence-based choice for reducing acute medication overuse headache among patients with chronic headache. Data from observational studies, clinical, and animal studies *in vivo* had confirmed the anti-migraine or analgesic effect of gastrodin but lack of convincible evidence supported by controlled trials. Acute withdrawal of overused medications is recommended by guidelines together with oral or subcutaneous prophylactic medication (27). The present two-arm placebo-controlled trial will investigate the efficacy of gastrodin in reducing monthly medication intake days, monthly headache frequency and improving health outcomes for people with MOH. We will also anticipate a higher acceptability and tolerability of gastrodin due to lower occurrence of serious adverse events brought by oral gastrodin in previous reporting.

Treatment options for withdrawal symptoms in the acute phase of detoxification vary considerably between studies including fluid replacement, analgesics, tranquilizers, neuroleptics, amitriptyline, valproate, intravenous dihydroergotamine, oxygen, electrical stimulation (46), diclofenac or naproxen orally, and/or metoclopramide, and behavioral therapies (47). Considering patients' clinical compliance to withdrawal treatment, bridging therapy is need other than specific symptomatic drugs. This hypothesis had been confirmed by a recently published 3-arm controlled trial (19). However, the best choice for bridging therapy is still debated. To avoid treatment failure to the most extent, we will introduce BI education (36) to all participants before randomization. The efficacy of BI scheme for MOH had been confirmed by a double-blinded cluster randomized controlled trial and recommended by a newly updated guideline (27). Taken together, we will look forward to imparting advice with restricted medication intake combined with oral gastrodin as a bridging therapy that may beneficial for patients with MOH with significant curative effect and better compliance.

A notable limitation of the present study protocol should be mentioned. Intranasal gastrodin was determined to be a promising alternative to the traditional administration route as gastrodin is not brain penetrant (33). Considering the lack of gastrodin nasal spray and there is no instruction for gastrodin overdosage in practice, we employ a placebo-controlled design in the present trial as a key approach to strengthen the probability of study success. Once the placebo effect of gastrodin in treating MOH is excluded by the present trial, future dose-effect studies will be implemented to explore the optimal dose of oral gastrodin in detoxification.

A well-tolerated, theoretically-based, and sustainable cessation of overused medication strategy is need. The present program holds potentials for providing new insights into administration of MOH in outpatient clinical care. In addition, we also intend to extend the current solution for withdrawal of overused acute medications with good tolerance and compliance.

## Ethics statement

The studies involving human participants were reviewed and approved by Ethic Committee of Affiliated Hospital of Yunnan University. The patients/participants provided their written informed consent to participate in this study.

## Author contributions

FK designed the trial and drafted the protocol manuscript. DB made conception and critical revisions of the work, mainly responsible for critical review on the trial design, and English language editing before and after submission. JG, JX, HL, and SM put forward suggestions on the revision of this manuscript. FK and JX were also responsible for replying to the reviewers' comments. All authors have provided approval for publication of the content and agreed to be accountable for all aspects of the work.
