# Supplementary material for: Efficacy and tolerability of oral gastrodin for medication overuse headache (EASTERN): Study protocol for a multicenter randomized double-blind placebo-controlled trial
Source: Front Neurol. 2023 Feb 22;13:1095298. doi: 10.3389/fneur.2022.1095298 (PMC9993247; doi:10.3389/fneur.2022.1095298)
Supplement: Supplementary file 1 [file Data_Sheet_1.docx]

Appendix. Diagnostic criteria of the ICHD-3 for medication overuse headache (MOH) and chronic migraine (CM)

MOH, code 8.2

Description: Headache occurring on 15 or more days/month in a patient with a pre-existing primary headache and developing as a consequence of regular overuse of acute or symptomatic headache medication (on 10 or more or 15 or more days/month, depending on the medication) for more than three months. It usually, but not invariably, resolves after the overuse is stopped.

Subtypes of MOH

8.2.1 Ergotamine-overuse headache: regular intake of ergotamine on ≥10 days/month for >3 months.

8.2.2 Triptan-overuse headache: regular intake of one or more triptans, in any formulation, on ≥10 days/month for >3 months.

8.2.3 Non-opioid analgesic-overuse headache: regular use of one or more non-opioid analgesics on ≥ 15 days/month for >3 months.

8.2.3.1 Paracetamol (acetaminophen)-overuse headache: regular intake of paracetamol on ≥ 15 days/month for >3 months.

8.2.3.2 Non-steroidal anti-inflammatory drug (NSAID)-overuse headache: regular intake of one or more non-steroidal anti-inflammatory drugs (NSAIDs) (other than acetylsalicylic acid) on ≥15 days/month for >3 months.

8.2.3.2.1 Acetylsalicylic acid-overuse headache: regular intake of acetylsalicylic acid on ≥15 days/month for >3 months.

8.2.3.3 Other non-opioid analgesic-overuse headache: regular intake of a non-opioid analgesic other than paracetamol or non-steroidal anti-inflammatory drugs (including acetylsalicylic acid) on ≥15 days/month for >3 months.

8.2.4 Opioid-overuse headache: regular intake of one or more opioids on ≥10 days/month for >3 months.

8.2.5 Combination-analgesic-overuse headache: regular intake of one or more combination analgesic medications on ≥10 days/month for >3 months.

8.2.6 Medication-overuse headache attributed to multiple drug classes not individually overused: regular intake of any combination of ergotamine, triptans, non-opioid analgesics and/or opioids1 on a total of ≥10 days/month for >3 months without overuse of any single drug or drug class alone.

8.2.7 Medication-overuse headache attributed to unspecified or unverified overuse of multiple drug classes: regular intake of any combination of ergotamine, triptans, non-opioid analgesics and/or opioids on ≥10 days/month for >3 months, the identity, quantity and/or pattern of use or overuse of these classes of drug cannot be reliably established.

8.2.8 Medication-overuse headache attributed to other medication: regular overuse, on ≥10 days/month for >3 months, of one or more medications other than those described above, taken for acute or symptomatic treatment of headache.

CM, code 1.3

Description: Headache occurring on 15 or more days/month for more than three months, which, on at least eight days/month, has the features of migraine headache.

Diagnostic criteria:

A. Headache (migraine-like or tension-type-like) on≥15 days/month for >3 months, and fulfilling criteria B and C

B. Occurring in a patient who has had at least five attacks fulfilling criteria B–D for 1.1 Migraine without aura and/or criteria B and C for 1.2 Migraine with aura

C. On ≥8 days/month for >3 months, fulfilling any of the following: 1. criteria C and D for 1.1 Migraine without aura; 2. criteria B and C for 1.2 Migraine with aura;3. believed by the patient to be migraine at onset and relieved by a triptan or ergot derivative; D. Not better accounted for by another ICHD-3 diagnosis.
